# A Potential Mechanism of Sodium Channel Mediating the General Anesthesia Induced by Propofol

**DOI:** 10.3389/fncel.2020.593050

**Published:** 2020-12-04

**Authors:** Jinglei Xiao, Zhengguo Chen, Buwei Yu

**Affiliations:** ^1^Department of Anesthesiology, Ruijin Hospital, Shanghai Jiao Tong University School of Medicine, Shanghai, China; ^2^College of Computer, National University of Defence Technology, Changsha, China

**Keywords:** propofol, voltage-gated sodium channels, Hodgkin–Huxley model, thalamocortical model, Hopf bifurcation, paradoxical excitation

## Abstract

General anesthesia has revolutionized healthcare over the past 200 years and continues to show advancements. However, many phenomena induced by general anesthetics including paradoxical excitation are still poorly understood. Voltage-gated sodium channels (Na_*V*_) were believed to be one of the proteins targeted during general anesthesia. Based on electrophysiological measurements before and after propofol treatments of different concentrations, we mathematically modified the Hodgkin–Huxley sodium channel formulations and constructed a thalamocortical model to investigate the potential roles of Na_*V*_. The ion channels of individual neurons were modeled using the Hodgkin–Huxley type equations. The enhancement of propofol-induced GABAa current was simulated by increasing the maximal conductance and the time-constant of decay. Electroencephalogram (EEG) was evaluated as the post-synaptic potential from pyramidal (PY) cells. We found that a left shift in activation of Na_*V*_ was induced primarily by a low concentration of propofol (0.3–10 μM), while a left shift in inactivation of Na_*V*_ was induced by an increasing concentration (0.3–30 μM). Mathematical simulation indicated that a left shift of Na_*V*_ activation produced a Hopf bifurcation, leading to cell oscillations. Left shift of Na_*V*_ activation around a value of 5.5 mV in the thalamocortical models suppressed normal bursting of thalamocortical (TC) cells by triggering its chaotic oscillations. This led to irregular spiking of PY cells and an increased frequency in EEG readings. This observation suggests a mechanism leading to paradoxical excitation during general anesthesia. While a left shift in inactivation led to light hyperpolarization in individual cells, it inhibited the activity of the thalamocortical model after a certain depth of anesthesia. This finding implies that high doses of propofol inhibit the network partly by accelerating Na_*V*_ toward inactivation. Additionally, this result explains why the application of sodium channel blockers decreases the requirement for general anesthetics. Our study provides an insight into the roles that Na_*V*_ plays in the mechanism of general anesthesia. Since the activation and inactivation of Na_*V*_ are structurally independent, it should be possible to avoid side effects by state-dependent binding to the Na_*V*_ to achieve precision medicine in the future.

## 1. Introduction

Anesthesia is considered one of the 10 greatest discoveries in medical history (Lawrence, 1999). The ideal state induced by general anesthesia is composed of three elements: antinociception, unconsciousness, and effective muscle relaxation. However, several unexpected phenomena have been reported in general anesthesia. First, some general anesthetics, typically propofol, could lead to a paradoxical excitation in light anesthesia with an incidence ranging from 14 to 70% (Jeong et al., 2011). Its clinical features are depicted as increased talkativeness, loss of cooperation, disorientation, excessive movement, sexual hallucinations, agitation, or rage, probably accompanied by increased activity in the higher beta-frequency bands (12.5–25 Hz) (Gugino et al., 2001; Jeong et al., 2011). Second, many clinical trials have shown that application of local anesthetics during general anesthesia decreases the usage of general anesthetics for maintenance of anesthesia (Senturk et al., 2002; Bazin et al., 2018). Potentiation of γ-GABAa receptor is a widely accepted molecular mechanism of general anesthetics, which results in an inhibition of the central nervous system (Yip et al., 2013; Jayakar et al., 2014). However, it cannot properly explain these unexpected phenomena. Further research may ensure better understanding of these phenomena to ensure the clinical safety of patients.

With the discovery of other targets, such as AMPA receptors, sodium channels, potassium channels, and calcium channels (Zhu et al., 1997; Ouyang et al., 2003; Liu et al., 2019), it has become apparent that general anesthetics affect multiple specific receptors (Franks and Lieb, 1984). Voltage-gated sodium channels (Na_*V*_) are the main channels which conduct the Na^+^ current inwardly, controlling the initiation and propagation of action potentials in nerves, muscles, and neuroendocrine cells. The Na_*V*_ is controlled by two gating mechanisms-activation and inactivation (Pan et al., 2018; Wang et al., 2018). Researches have shown that general anesthetics modulate the gating parameters of Na_*V*_. Propofol directly interacted with rat brain Na_*V*_ and suppressed the inward Na^+^ flux with a plasma concentration from 10 to 50 μM (Rehberg and Duch, 1999). In Ouyang' research, 5 μM propofol shifted both activation and inactivation parameters of Na_*V*_, while 0.8 mM isoflurane produced a left shift in inactivation (Ouyang et al., 2003). Another study showed that ketamine induced a concentration-dependent left shift in both activation and inactivation in human neuronal Na_*V*_ with a maximum shift of 5 mV (Reckziegel et al., 2002). However, the effects of the interactions between anesthetics and Na_*V*_ during general anesthesia are still poorly understood.

In this study, we measured the influences of different concentrations of propofol on the gating parameters, both activation and inactivation, of bacterial Na_*V*_ (NaChBac) by using electrophysiology experiments, and then modified the Hodgkin–Huxley(H-H) Na_*V*_ formulations and constructed a thalamocortical model to further understand the dynamics of Na_*V*_ under general anesthesia. The electrophysiology results indicated that the influence of propofol on activation and inactivation occurred at different concentrations: propofol caused a left shift on Na_*V*_ activation with concentrations from 0.3 to 20 μM, while a left shift in inactivation increased with increasing concentration (0.3–30 uμM). As propofol obviously increased Na^+^ flux at low concentrations (1 and 3 μM) while decreased it at high concentrations (20–30 μM), it implied that light anesthesia primarily accelerates Na_*V*_ activation and deep anesthesia enhances Na_*V*_ inactivation. By mathematically fitting the electrophysiology data to an H-H neuron model, we confirmed that a left shift in activation produced spontaneous oscillations at Hopf bifurcations. While the left shift of Na_*V*_ reached a certain value, around 5.5 mV, the normal bursting of TC cells was suppressed by triggering its chaotic oscillations. This led to irregular spiking of PY cells and an increased frequency in EEG readings. This finding suggested a dynamic mechanism for paradoxical excitation during anesthesia induction. The left shift of inactivation parameters decreased the firing rate after a certain depth of general anesthesia. This observation indicated that high doses of propofol inhibited the network partly by enhancing inactivation, and provided one explanation of why the application of lidocaine decreases the required dosage of a general anesthetic. Our results revealed the different effects of Na_*V*_ activation and inactivation observed in general anesthesia. Further, it provided an insight into the possible roles of the involved proteins in general anesthesia, in combination with the other targeted proteins. Therefore, developing Na_*V*_ state-dependent anesthetics may likely decrease the incidence of side-effects and aid in precision medicine by avoiding the effects of Na_*V*_ activation.

## 2. Methods

### 2.1. Cell Culture and Transfection

NaChBac cDNA constructed in a modified *pLenti6-CMV*_2_ expression vector, was amplified in *E.coli* C3040. *HEK 293* cells were transiently transfected using Polyethylenimine (Polyscineces) in 48 h before recording, and cultured in Dulbecco's modified Eagle's medium/F-12 (Invitrogen) with 5% (v/v) fetal bovine serum (BioSource International, Camarillo, CA) and 5% CO_2_, 95% O_2_ (v/v) at 37°C.

### 2.2. Electrophysiology

Whole-cell patch clamping was used to measure the responses of sodium channels to propofol of different concentrations. Extracellular solution (ESC) contained (in mM): 140 NaCl, 4 KCl, 1.5 CaCl_2_, 1.5 MgCl_2_, 10 Hepes and 5 D-glucose, pH 7.4 adjusted with NaOH, Osmolality was 315 mOsm/kg. Intracellular solution contained (in mM): 15 NaCl, 80 CsF, 40 CsCl, 10 EGTA, and 10 Hepes, pH 7.3 adjusted with CsOH, Osmolality was 290 mOsm/kg. The stock solution consisted of 200 mM propofol dissolved in DMSO. The stock solution was diluted to working concentrations with ECS. All dilutions were prepared with a concentration of 0.05% DMSO. Following control recordings, cells were perfused with propofol for 2 min before the collection of paired anesthetic recordings, and each cell was used for only one concentration. The activation protocol was as follows: 200 ms depolarizing steps (from −100 to +60 mV, ΔV = 10 mV) with a holding potential of −100 mV. The inactivation protocol involved a 2 s conditioning pulse (−120 mV to 0 mV, ΔV = 10 mV), followed immediately by a 50 ms test pulse to −20 mV; holding potential was −100 mV. Clampfit 10.7 and Graphpad were used to analyze the voltage-clamp data. All parameters evaluated are reported as mean ± SEM. Paired t-tests were used to assess the statistical significance of differences between paired data sets in the absence and presence of propofol; a p value of 0.05 was considered to be significant. The conductance (G) value was calculated as

(1) $$\begin{array}{l} G=I_{Na}/\left(V-V_{r}\right) \end{array}$$

where *I*_*Na*_ is peak Na^+^ current, *V* is test potential, and *V*_*r*_ is reversal potential. According to the definition of steady-state of activation and inactivation, $m_{\infty }^{3}$ is equal to the normalized *G* by *G*_*max*_, while *h*_∞_ is equal to the normalized *I* by *I*_*max*_ under the inactivation protocol. The voltage dependence of *m*_∞_ or *h*_∞_ were fit to Boltzmann function:

(2) $$\begin{array}{l} X_{\infty }\left(V\right)=1/\left[1+e^{\left(V_{50}-V\right)/k}\right] \end{array}$$

where *V*_50_ is the midpoint voltage of the activation, and *k* is the slope factor. The time constants of activation (τ_*a*_) and inactivation (τ_*in*_) were derived from the rising and decaying components of the Na^+^ current respectively, by fitting to a single exponential function:

(3) $$\begin{array}{l} I\left(t\right)=Ae^{t/\tau }+C \end{array}$$

where *A* is the amplitude, *C* is the plateau constant, *t* is time, and τ is the time constant of activation or inactivation.

### 2.3. Parameter Estimation

According to the electrophysiology results of activation and inactivation, we got the values of *m*_∞_ and *h*_∞_ at corresponding voltages as well as the τ_*a*_ and τ_*in*_. We fitted the results to the following equations to get the values for gating parameters:

(4) $$\begin{array}{l} x_{\infty }\left(V\right)=\alpha /\left(\alpha +\beta \right) \end{array}$$

(5) $$\begin{array}{l} \tau \left(V\right)=1/\left(\alpha +\beta \right) \end{array}$$

(6) $$\begin{array}{l} \alpha_{m}\left(V\right)=a\left(V+c\right)/\left(1-e^{-\left(V+c\right)/b}\right) \end{array}$$

(7) $$\begin{array}{l} \beta_{m}\left(V\right)=ae^{-b\left(V+c\right)} \end{array}$$

(8) $$\begin{array}{l} \alpha_{h}\left(V\right)=ae^{-b\left(V+c\right)} \end{array}$$

(9) $$\begin{array}{l} \beta_{h}\left(V\right)=a/\left(1+e^{-b\left(V+c\right)}\right) \end{array}$$

The fitted results under extracellular solution are shown in Table 1 MATLAB (R ≥ 0.90). Based upon the electrophysiology results, we added the corresponding shifts in α_*m*_/β_*m*_ and α_*h*_/β_*h*_ to simulate the propofol effects.

**Table 1:** Fitting results from electrophysiology.

**Parameters**	**α_*m*_**	**β_*m*_**	**α_*h*_**	**β_*h*_**
a	3.5	0.6	0.1	5
b	4	0.06	0.08	0.15
c	55	0	15	57
R-square	0.99	0.98	0.95	0.92

### 2.4. Model

Each cell/compartment was modeled as a set of ordinary differential equations, summarized as:

(10) $$\begin{array}{l} \dot{V}=C\left(-\sum I_{memb}-\sum I_{syn}+I_{app}\right) \end{array}$$

**Figure 1:**
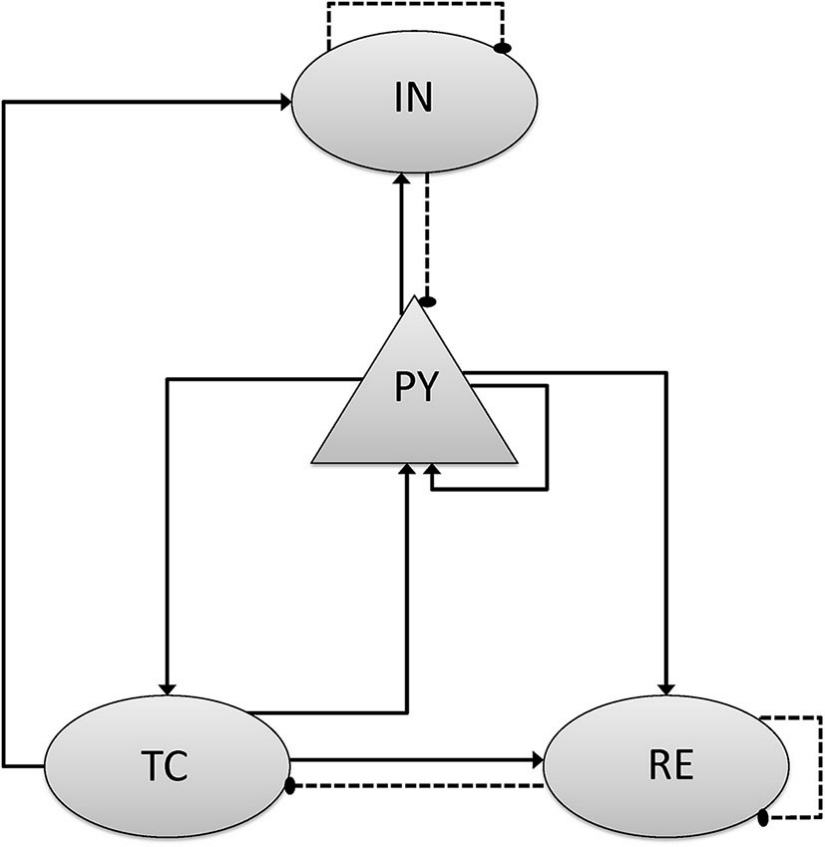
Scheme of network model and connection mechanism. The network consists of separate populations of pyramidal cells (PY), Inhibitory cells (IN) that interact with thalamic reticular cells (RE) and thalamic relay cells (TC). The triangular arrow with full line stands for the excitatory synaptic connection with AMPA mechanism, and the circle one with dotted line stands for the inhibitory synaptic connection with GABAa mechanism.

*I*_*memb*_ and *I*_*syn*_ stand for the membrane and synaptic currents respectively, and determine the dynamics of each type of neuron. *I*_*app*_ denotes external applied currents specifically to each cell. Each current is governed by differential equations representing biophysiology, based on the Hodgkin–Huxley formulations (Pospischil et al., 2008; Ching et al., 2010), in which the transmembrane currents for each cell determine its dynamic behavior. The following subsections provided an overview of these currents. Further details and equations are provided in the Supplementary Methods. In the model, the cortical-thalamus network was composed of 400 PY cells, 80 IN cells, 80 TC cells and 80 RE cells. Simulations were carried out using MATLAB DynaSim (Sherfey et al., 2018). PY and TC cells propagated with AMPA receptors, while IN cells and RE cells with GABAa receptors. As the delay between cortex and thalamus is described in biological observation (Hashemi et al., 2017), the delay from thalamus to cortex was set as 20 ms, while from cortex to thalamus was set as 60 ms. Synaptic connection between each cells were modified according to the results of previous studies (Figure 1 and Table 2) (Destexhe et al., 1998; Ching et al., 2010; Hayut et al., 2011; Fogerson and Huguenard, 2016; Krishnan et al., 2016).

**Table 2:** The synaptic connection between cells.

	**Propagation**	**Radius**	**g_*GABA*_*a*__ (mS/cm^2^)**	**g_***AMPA***_ (mS/cm^**2**^)**
PY	PY	5	/	0.1
	IN	1	/	2
	RE	8	/	0.1
	TC	10	/	0.1
IN	PY	5	2	/
	IN	2	1	/
TC	PY	10	/	0.1
	IN	2	/	2
	RE	8	/	0.1
RE	TC	8	0.06	/
	RE	5	0.06	/

Cortical cells: Our model included PY and IN cells. Cells contained fast sodium (*I*_*Na*_), potassium (*I*_*K*_), and leak (*I*_*Leak*_) currents which are essential for spiking. A slow potassium current (*I*_*M*_) was included in the model of the PY cells. The applied current (*I*_*app*_) determined the specific baseline firing rate of a given cell.

Thalamic cells: TC and RE cells were included in Thalamus, which also contained essential currents. TC cells had a T-type calcium current (*I*_*T*_) and *I*_*h*_. The RE cells also contained *I*_*T*_.

Synaptic connectivity (*I*_*syn*_): The synaptic connections in the brain are complicated, and includes roles for AMPA receptor, GABAa receptor, NMDA receptor, and glutamate receptor, amongst others. To simplify the simulation, only excitatory AMPA (*I*_*AMPA*_) and inhibitory (*I*_*GABAa*_) were included. We simulated the propofol effects on GABAergic neuron through an increase in the conductance and decay time (Ching et al., 2010; Flores et al., 2017).

Applied current and noise: These parameters consisted of a constant drive, plus a train of excitatory postsynaptic potentials generated using a Poisson process. The applied current simulated all exogenous inputs, not explicitly included in the model, to each cell. The constant drive was chosen randomly from a uniform distribution. The background drive was applied only to cortical cells.

Estimation of the models: The EEG was modeled as average of post-synaptic potential from PY cells (Ching et al., 2010). To partly estimate consciousness, we randomly combined the average firing rate of 400 individual cells into a 400*400 matrix, an approach which was inspired by a simulation study, which suggests that consciousness could be related to binding neurons into cliques with increasingly higher dimension to represent features of the stimulus (Reimann et al., 2017). The output of each cell was a pixel in an image, as shown in Zhou's study (Zhou et al., 2015). FR is the average firing rate of one certain population cells in 5 s. CV is its coefficient of variation. Models were simulated and analyzed using wavelet analysis by MATLAB with fixed-step solvers, with a maximum time step of 0.02 ms.

## 3. Results

### 3.1. The Effects of Propofol on the Activation and Inactivation of Na_*V*_ Showed Differences in Concentration Dependence

**Figure 2:**
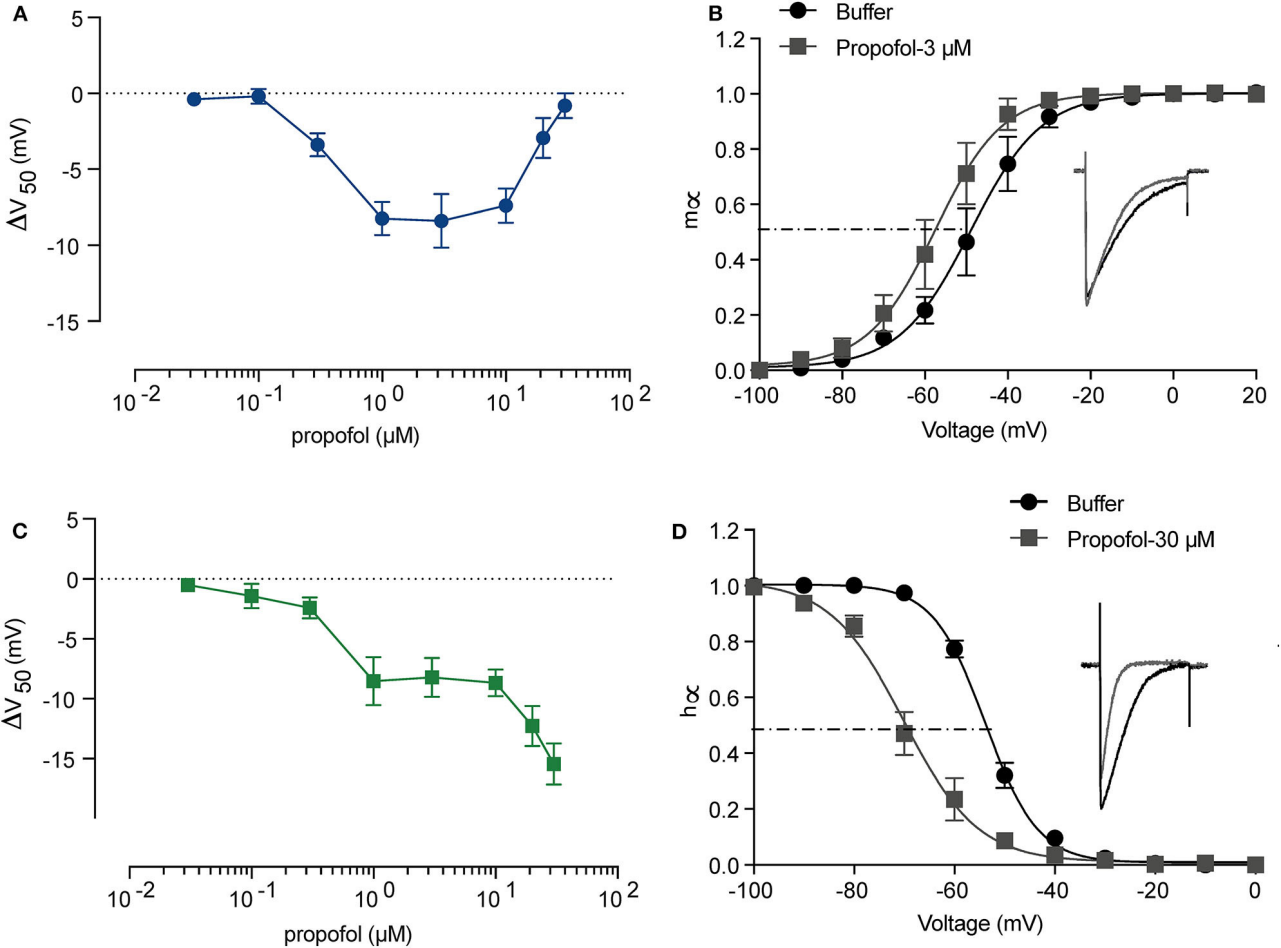
The activation and inactivation of sodium channels (NaChBac) showed different responses to propofol. A summary of activation **(A)** and inactivation **(C)** shifts induced by propofol with gradient concentration. **(B)** Activation *m*_∞_V curves under 3 μM propofol and a comparison of the traces at 0 mV. **(D)** Inactivation *h*_∞_V curves under 30 μM propofol and a comparison of the traces at 0 mV. *m*_∞_V and *h*_∞_V curves were fitted to Boltzmann equation. The data are shown as mean ± SEM, and paired *t*-test was applied. *n* = 5–8.

It has been reported that the EC_50_ of propofol in human plasma is 11–17 μM (2.0–3.14 μg/mL) (Kodaka et al., 2004; Li et al., 2015) and deep anesthesia requires 28–34 μM (5–6 μg/mL) (Casati et al., 1999). To investigate the manner in which different concentrations of propofol affect Na_*V*_, we established a concentration gradient with 0.03, 0.1, 0.3, 1, 3, 10, 20, and 30 μM propofol, and measured corresponding activation and inactivation parameters by electrophysiology. Supplementary Figure 1 shows the trace of NaChBac at different voltages, and the curves of the corresponding parameters. When treated with ECS, NaChBac was activated by a certain range of voltage (−70 to 60 mV). After reaching to a peak current, the channel started to be inactivated. Once the inward Na^+^ influx is equal to the outward current, it is the reverse voltage (*V*_*r*_), which was 60 mV. After 2-min perfusion with propofol, the activation showed an increasing left shift from 0.3 μM, reaching a maximum shift at 3 μM (Figure 2B), and then decreasing to be barely evident at 30 μM (Figure 2A and Table 3). Inactivation exhibited an increasing left shift with increasing concentration (0.3–30 μM) (Figure 2C and Table 3), with a maximum shift at 30 μM (Figure 2D). Enhancement of activation is mainly induced by low concentrations of propofol, and acceleration of inactivation takes place in a dose-dependent manner. As shown in the IV curve, when the propofol tremendously affects activation, peak current of *Na*_*V*_ is increased compared with the control results, implying that a low concentration of propofol should have a positive influence on sodium channels (Supplementary Figure 2A and Table 3). When the propofol concentration primarily left shifted inactivation, peak current of *Na*_*V*_ was decreased compared with the control results, implying that high concentrations of propofol inhibited sodium channels (Supplementary Figure 2B and Table 3).

**Table 3:** A summary of the changes in Na_*V*_ under different concentrations of propofol.

**Propofol (uM)**	**Δm (mV)**	**Δz_***a***_**	**Δh (mV)**	**Δz_***in***_**	**ΔA**	**ΔV_***r***_ (mV)**
0.03	0.4 ± 0.16	0.3 ± 0.25	0.5 ± 0.47	0.5 ± 0.57	0.08 ± 0.044	0.5 ± 0.43
0.1	0.2 ± 0.48	0.1 ± 0.26	1.4 ± 1.03	-1.6 ± 1.56	0.06 ± 0.036	1.3 ± 1.15
0.3	3.4 ± 0.76^*^	0.2 ± 0.24	2.4 ± 0.87^*^	1.0 ± 0.74	0.10 ± 0.020	1.7 ± 2.08
1	8.2 ± 1.10^**^	0.5 ± 0.64	8.5 ± 2.00^**^	-1.3 ± 1.47	0.17 ± 0.029^**^	1.4 ± 2.08
3	8.4 ± 1.77^**^	0.2 ± 0.74	8.2 ± 1.62^**^	0.4 ± 0.47	0.17 ± 0.058^*^	1.0 ± 1.91
10	7.4 ± 1.15^**^	0.8 ± 0.63	8.7 ± 1.11^**^	-0.2 ± 0.80	0.04 ± 0.032	1.1 ± 1.20
20	2.9 ± 1.31	0.6 ± 0.33	12.3 ± 1.67^**^	1.0 ± 0.63	-0.04 ± 0.039	0.2 ± 1.81
30	1.8 ± 0.40	0.6 ± 0.4	15.4 ± 1.73^***^	1.1 ± 0.37^*^	-0.14 ± 0.046^*^	1.5 ± 1.23

Δ*m is the shift of activation, and* Δ*h is the shift of inactivation. The positive value stands for left shift, and the negative stands for right shift.* Δ*z_a_ and* Δ*z_in_ were the changes of z in activation and inactivation respectively.* Δ*A was the changes of the normalized maximum amplitude.* Δ*V_r_ was the changes of the reverse voltage. z = 26/slope*.

* *p < 0.05*,

** *p < 0.005*,

*** *p < 0.0005*.

### 3.2. The Modified H-H Sodium Channel Model Fitted the Biological Data

**Figure 3:**
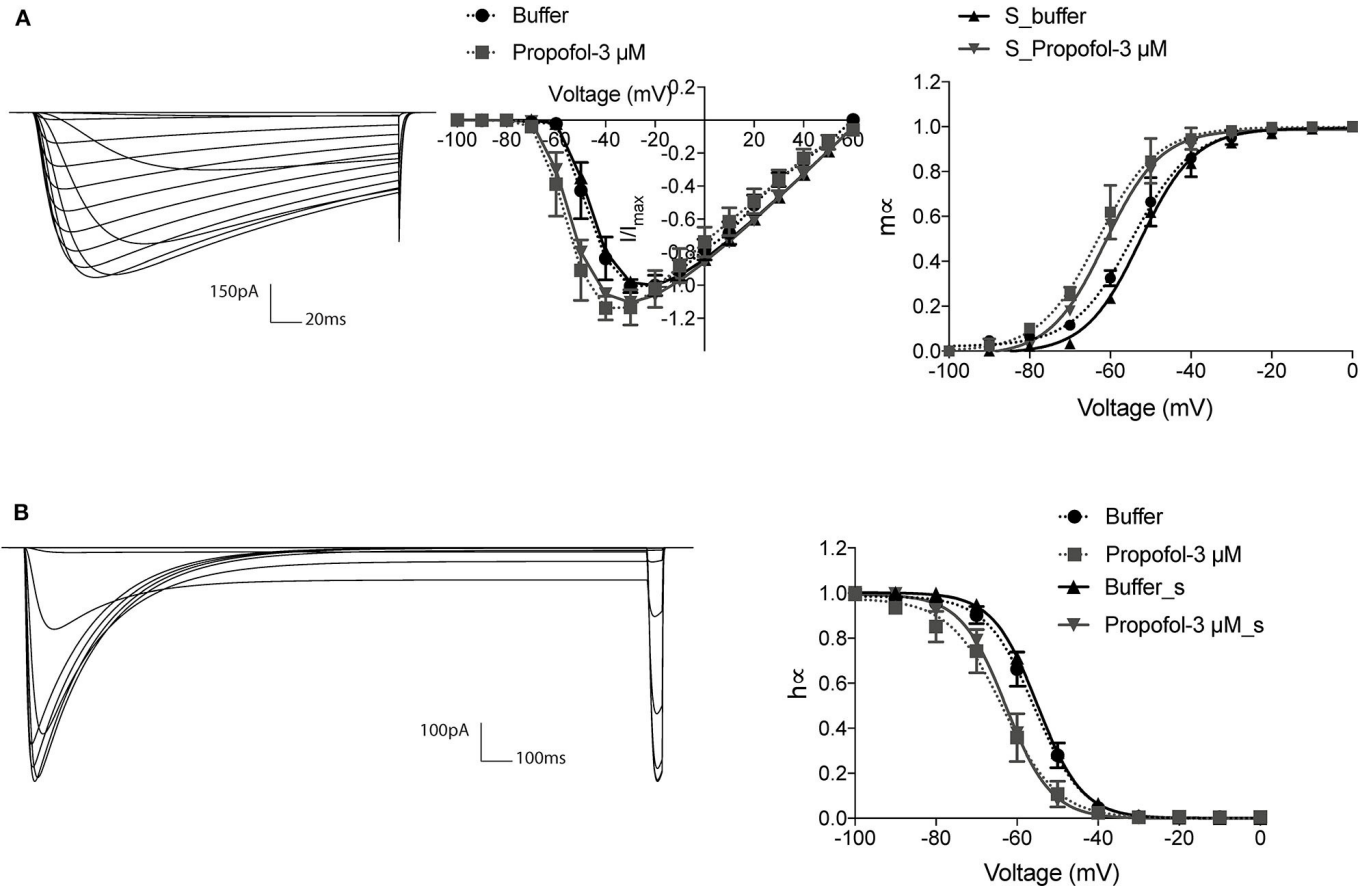
The modified H-H sodium channel model fitted the biological data. The current traces and corresponding gating curves from simulation results under activation **(A)** and inactivation **(B)** protocols, compared with electrophysiology data of 3 μM propofol. m_∞_V and h_∞_V curves were fitted to Boltzman equation. The electrophysiology data was shown as means ± SEM, and paired *t*-tests were applied. *n* = 6–7.

To further understand the possible dynamic changes induced by the propofol effects on sodium channel, we established a method to simulate these effects based on H-H equations. As the electrophysiology results showed that propofol led to a left shift in activation or inactivation, with no significant changes in either *V*_*r*_ or *k* (Table 3), we simulated the left shift of *V*_50_ by left shifts in the equations of α_*m*_ and β_*m*_, and α_*h*_ and β_*h*_, in accordance with the H-H model equations. Because a slightly increasing inactivation slope was only found at concentrations of 30 μM, the change of slope was not considered. We acquired traces and the corresponding curves of activation (Figure 3A) and inactivation (Figure 3B) using IonChannelLab (De Santiago-Castillo et al., 2010). The simulated gating parameter curves (black solid curves) were matched with the electrophysiology results (black dotted curves). Then, we applied the propofol effects to the model. As expected, in the simulation results corresponding to the effects of 3 μM propofol, *m*_∞_V and *h*_∞_V curves calculated from the model with corresponding shifts of α_*m*_(*V*), β_*m*_(*V*), α_*h*_(*V*), and β_*h*_(*V*) (gray solid curve) fitted the data we obtained from the electrophysiology experiment, as well as the normalized IV curve (Figure 3, gray dotted curve). These results demonstrated that the effects of propofol on sodium channels could be simplified to modify the equations of α(V) and β(V) to α(V+ΔV_50_) and β(V+ΔV_50_).

### 3.3. The Acceleration of Na_*V*_ Activation Produced a Limited Oscillation

**Figure 4:**
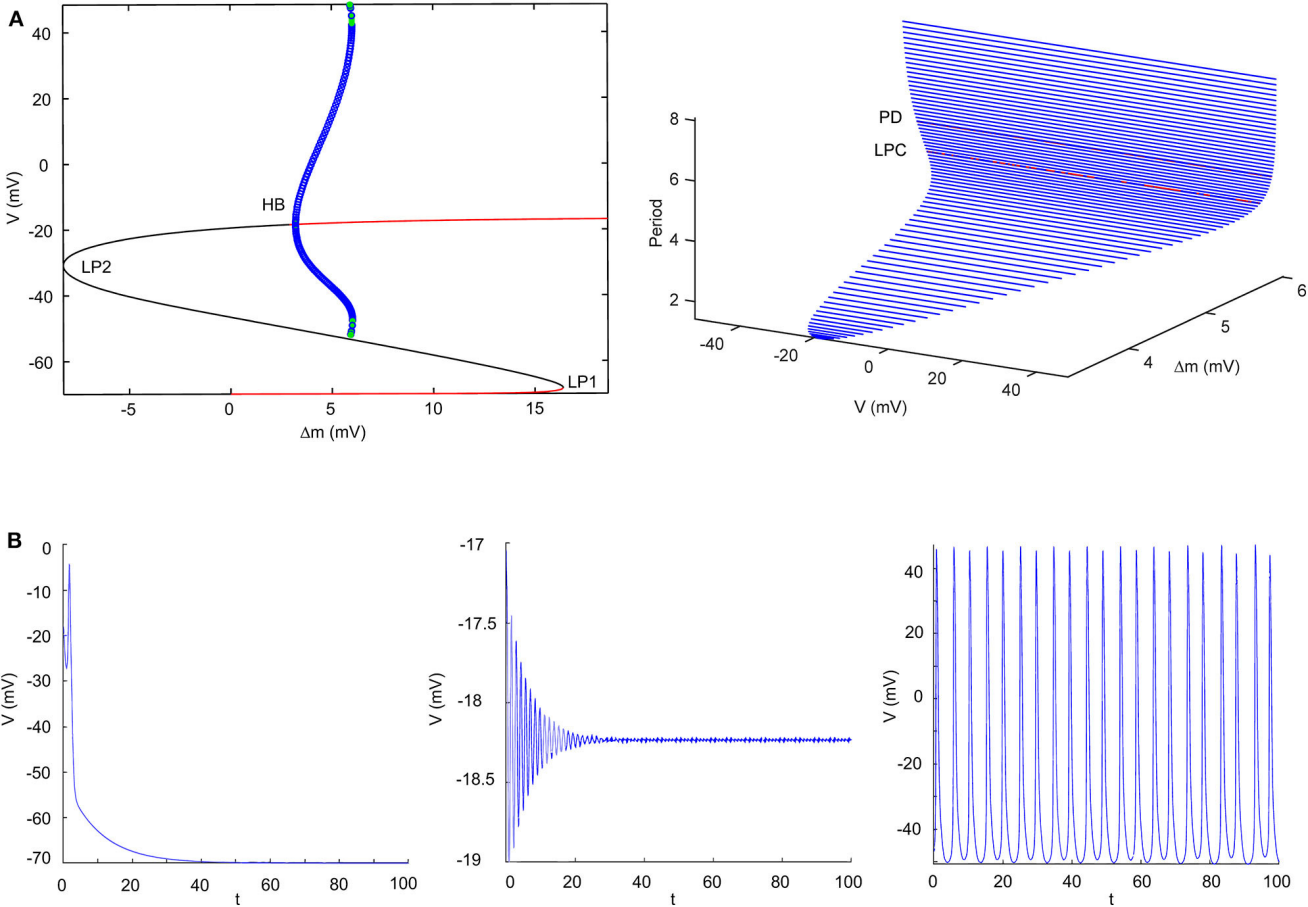
Results of single parameter bifurcation analysis. **(A)** The bifurcation diagram of one parameter bifurcation analysis by varying Δm (left), and period changes with varying Δm (right). **(B)** The activities of the cell model with an initiation state of V_0_ = -15 mV with Δm = 0 mV (Left), Δm = 3.2 mV (middle, Hopf point), Δm = 5.9 mV (right, LPC) both with I_*app*_ = 0. LP1 and LP2 are the limited points, HP is the Hopf bifurcation point, LPC is the Limited Point of Cycle, and PD is period doubling bifurcation.

**Figure 5:**
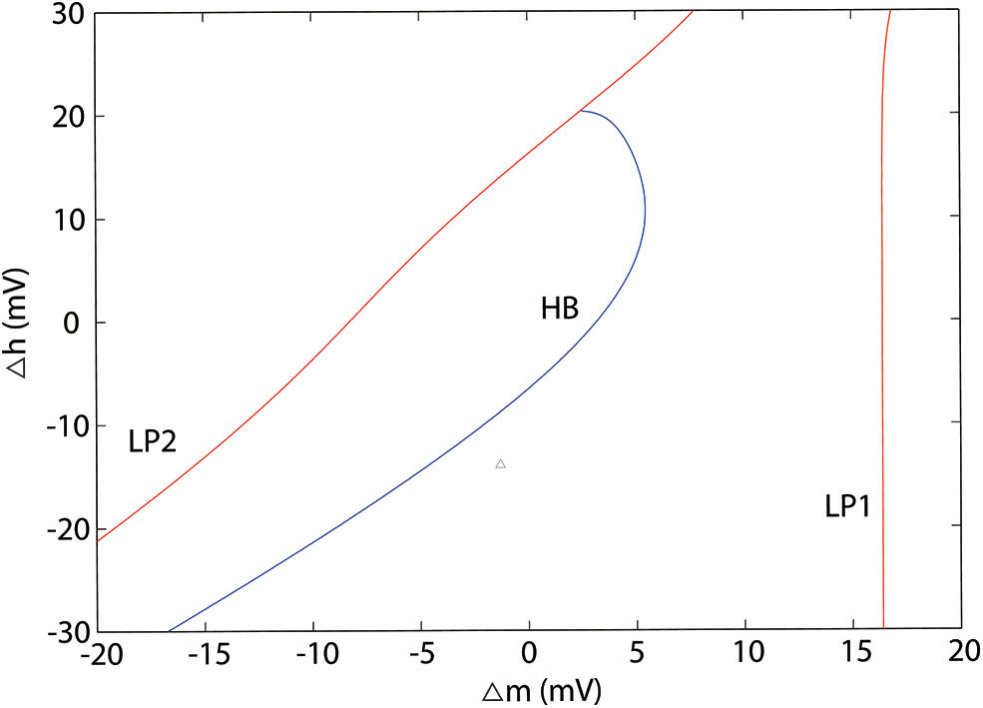
Two parameters bifurcation analysis of Δm and Δh. LP1 and LP2 are the limited points, HP is the Hopf bifurcation point.

According to the modified H-H sodium channel models, we analyzed the dynamic changes in a single neuron that propofol may lead to. In general, sodium and potassium channels are the two elements essential for producing an action potential for most neurons. To investigate the effects of shifts in activation (Δm) and inactivation (Δh), we used a neuron cell model incorporating biological parameters based on the H-H model, which included *I*_*Na*_, *I*_*K*_, and *I*_*leak*_ (Pospischil et al., 2008). For the dynamic system calculations, we used MatCont (Dhooge et al., 2003) or Xpp (Ermentrout, 2002) to determine the solutions of the ODEs. In the model (Figure 4B, left) without shifts in activation or inactivation, the resting potential (*V*_*rest*_) was −70 mV. In the bifurcation diagram, increasing Δh lightly hyperpolarized the cell model (**Figure 11A**), while there was a Hopf bifurcation and limited cycles with a varying Δm (Figure 4A and Table 4). The period of oscillation was positively correlated to Δm. It indicated that increasing left shit of Na_*V*_ activation led to a limited oscillation (Figure 4B, middle and right), a phenomenon which was expected to revert to *V*_*rest*_ gradually without shift in Δm (Figure 4B, left). These observations indicate that left shift in Na_*V*_ activation increased individual neuron activity. Per our electrophysiology results, propofol also led to a left shift in inactivation. Hence, we performed two parameters bifurcation analysis to verify whether the left shift of inactivation could affect the oscillation (Figure 5). Increasing left shift of Na_*V*_ inactivation led to a right shift of Hopf bifurcation point, which postponed the spontaneous oscillation induced by left shift of Na_*V*_ activation. The two parameters analysis also indicated that oscillation burst at Hopf bifurcation existed while Δh is less than 20.5 mV. The results of our electrophysiology measurement indicated that acceleration of Na_*V*_ activation induced by low concentrations of propofol could lead to oscillatory activities.

**Table 4:** The special point of the bifurcation diagram with varying V_*m*_.

**Par**	**V**	**m**	**h**	***n***	**Δm**
LP1	−68.25	0.028	0.999	0.003	16.4
LP2	−30.74	0.211	0.342	0.470	−8.3
Hopf	18.42	0.857	0.031	0.706	3.2

*LP, Limit point; Hopf, Hopf bifurcation*.

### 3.4. Interaction Between Propofol and Sodium Channels Induced a Paradoxical Excitation in Thalamocortical Models

**Figure 6:**
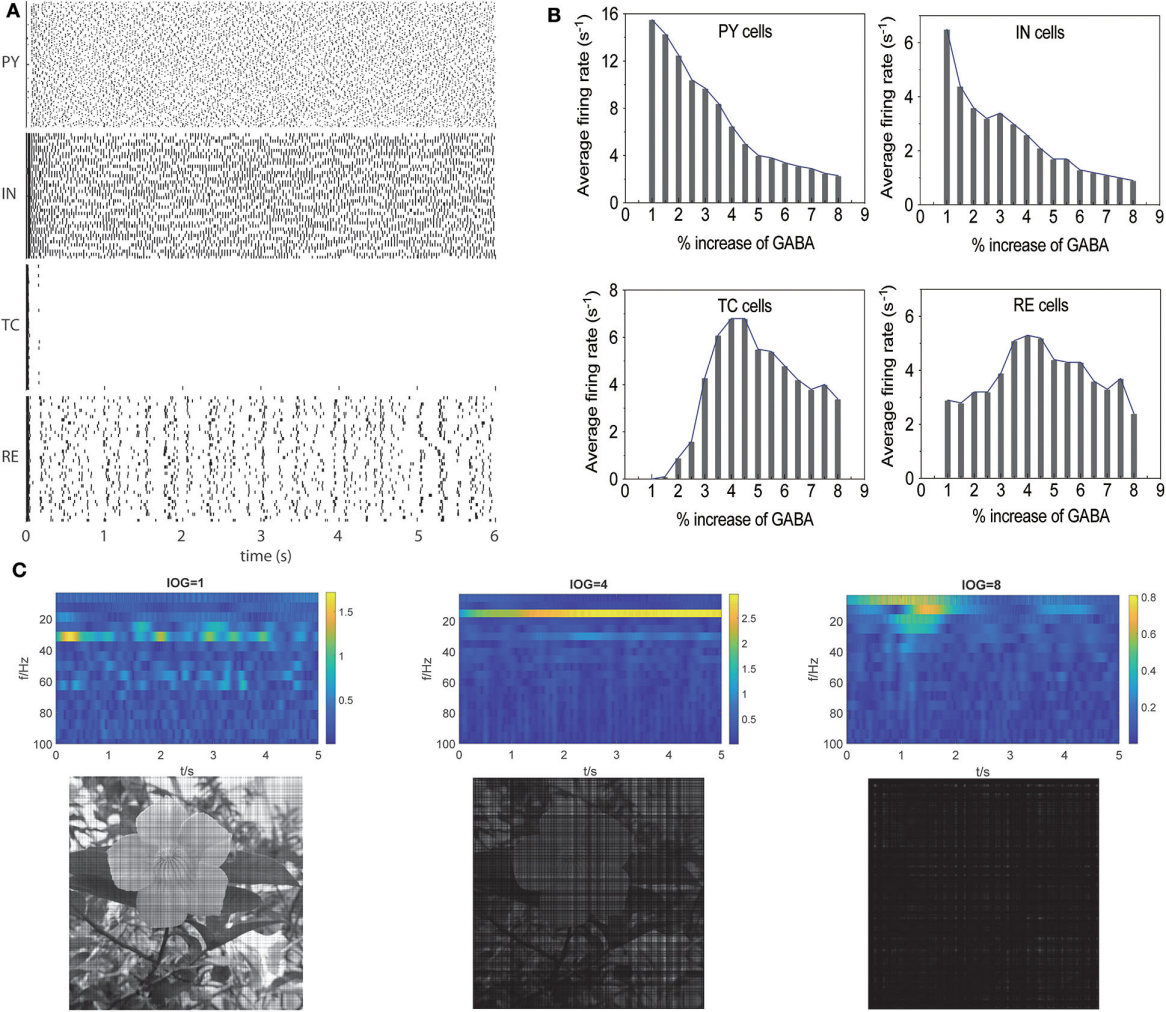
Propofol potentiating GABAa receptor in thalamocortical models induced unconsciousness. **(A)** Activities of different cell populations at awake status. **(B)** Average firing rate (>-20 mV) in all the cell populations with increasing concentration of propofol. **(C)** Evaluation of our models using the results of EEG (the first lanes), consciousness (the second lane) at *IOG* value of 1, 4, 8.

As low concentration of propofol can lead to oscillatory activities in individual cells, the activity of neurons in a network is more complicated for various synaptic propagations. To investigate whether the dynamic changes in individual neurons induced by interactions between propofol and Na_*V*_ would affect the brain network, we constructed a thalamocortical model (Figure 1). Cells activities at awake state are shown in Figure 6A. PY, IN, and RE cells displayed active spiking, while TC cells barely registered spiking, which is consistent with previous publication (Ching et al., 2010). In the awake state (with increase of GABAa, IOG of 1), the model was characterized by a predominance of frequencies in the β (15–30 Hz) and γ (30–80 Hz) ranges, similar to the awake brain features described in previous studies (Bressler, 1990; Freeman, 1991). To establish the relationship of γ-GABA_*a*_ receptors and the depth of anesthesia, we simulated the effects of propofol on γ-GABA_*a*_ receptors by enhancing the conductance and the time constants as reported in Flores's study (Flores et al., 2017). As studies demonstrated amplitude deepening in EEG waveforms and lower EEG frequency emerging with the deepening of general anesthesia (Hagihira, 2015), the α (7–14 Hz) wave was related to loss of consciousness (equal to EC_50_ concentration of clinical propofol), and the δ (0.5–3.5 Hz) wave showed a typical EEG manifestation of deep anesthesia (Ching et al., 2010) with total unconsciousness. These are important indices to evaluate the relation of *IOG* and consciousness. Thus, we examined our models to check whether the PY cells activities, consciousness outputs, and EEG results met the clinic observation. Our simulation results showed that with increasing propofol concentration, cortical activities in general gradually decreased (Figure 6B). The thalamus cell activities were increased at light anesthesia, and then decreased after a 4-time *IOG* that was related to the onset of spindle-band activity (Urbain et al., 2019). Moreover, the frequency of EEG recording decreased with *IOG* from 1 to 8 (Figure 6C). With an *IOG* of 8, the activity of PY cells showed predominate frequencies in the δ range (0.5–3.5 Hz). Further, our study identified complete unconsciousness at an *IOG* value of 8 (Figure 6C). We therefore adopted *IOG* 8 to simulate the deep anesthesia state, corresponding to a maintenance period of 30 μM propofol (Casati et al., 1999).

**Figure 7:**
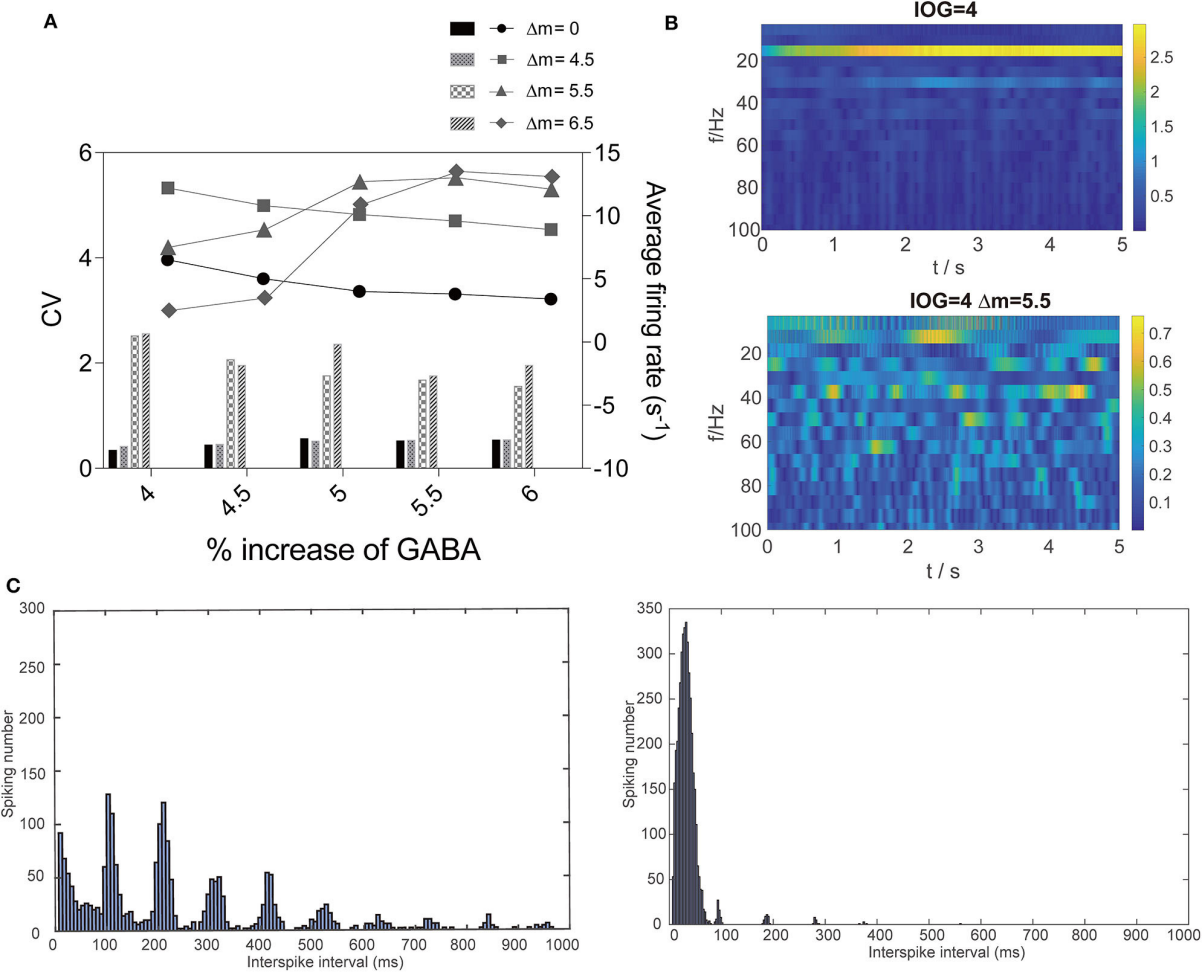
Dynamic changes that interaction of propofol and Na_*V*_ led to. **(A)** Comparison of average firing rate and CV of firing rate with or without the effect on Na_*V*_ with Δm = 4.5, 5.5, and 6.5 mV in PY cells. **(B)** EEG and **(C)** spiking interval changes at *IOG* value of 4 within (right) or without (left) effect on Na_*V*_.

Then, we studied the dynamic changes that propofol accelerating Na_*V*_ activation and inactivation led to while propofol also potentiated γ-GABA_*a*_ receptor. We found that when the left shift of Na_*V*_ activation was less than 5.5 mV, it mainly increased PY cells' firing rate. However, when the left shift of Na_*V*_ activation was over 5.5 mV, the CV of PY cells were also increased (Figure 7A). At these points, such as at *IOG* values of 4, the dominant EEG frequency significantly increased with the emerging of β and γ waves (Figure 7B). As shown in Figure 7C, most cells spiking intervals were in the range of 0-100 ms, which revealed uniformed and fast spiking in these cells. As Destexhe described that increasing CV and fast but more uniformed cortical cells spiking are the features of asynchronous irregular states in the thalamocortical network (Destexhe, 2009), our results claimed a irregular state which fit the clinic features of paradoxical excitation that patients are with cognition dysfunction and increased EEG dominant band. This part implies a dynamic mechanism leading to paradoxical excitation.

### 3.5. Hopf Bifurcation Induced by Na_*V*_ Activation in TC Cells Triggered Irregular Activity of Thalamocortical Models

**Figure 8:**
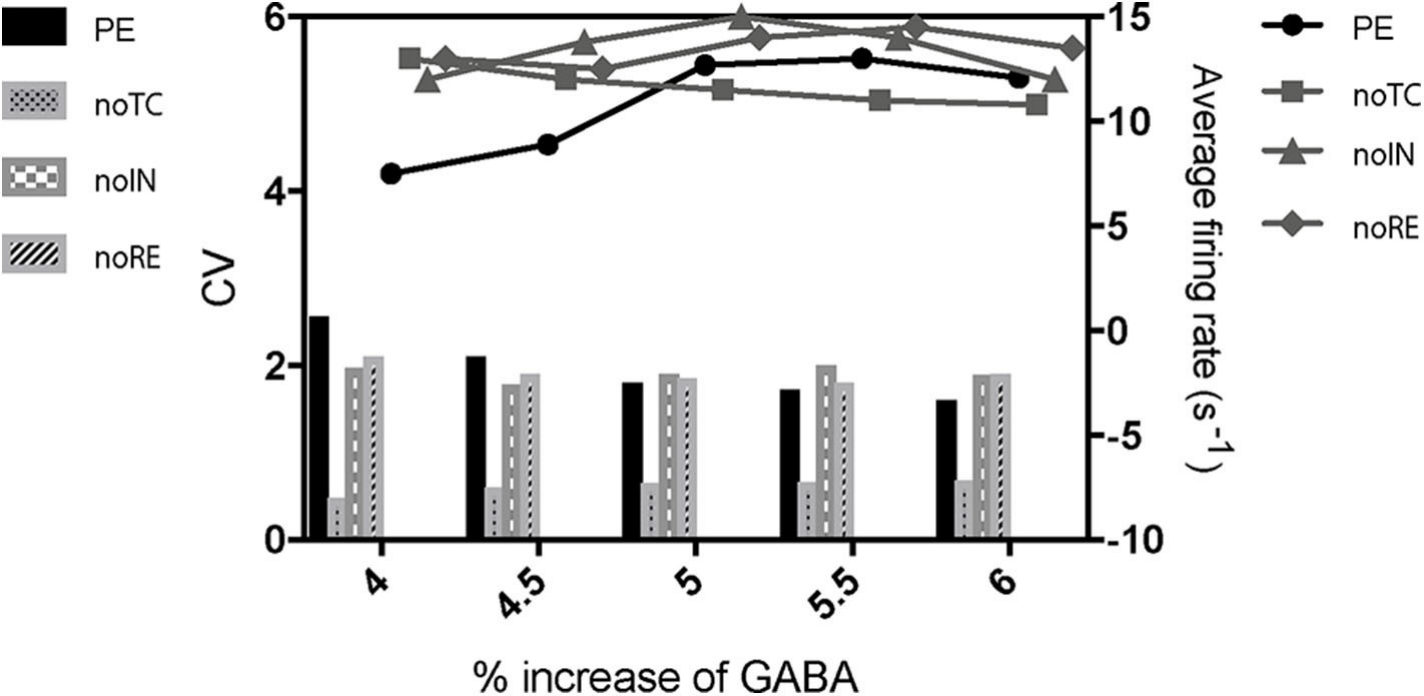
The effects of cell populations on the paradoxical phenomenon. PE means paradoxical excitation. noTC, noIN, and noRE mean manually removing the left shift of Na_*V*_ activation in corresponding cell populations.

**Figure 9:**
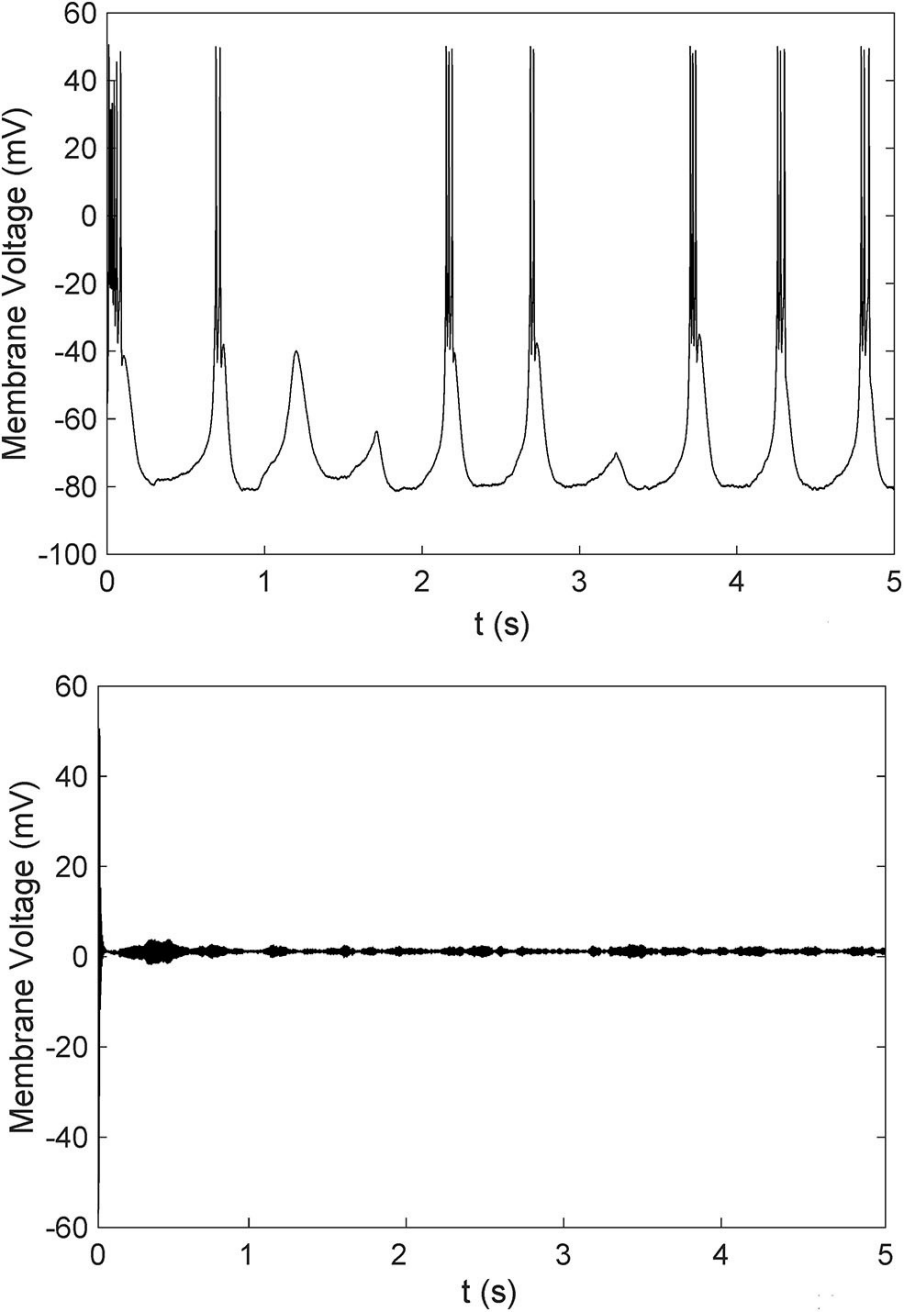
A comparison of cells spiking of TC cells with **(bottom)** or without **(top)** paradoxical excitation.

**Figure 10:**
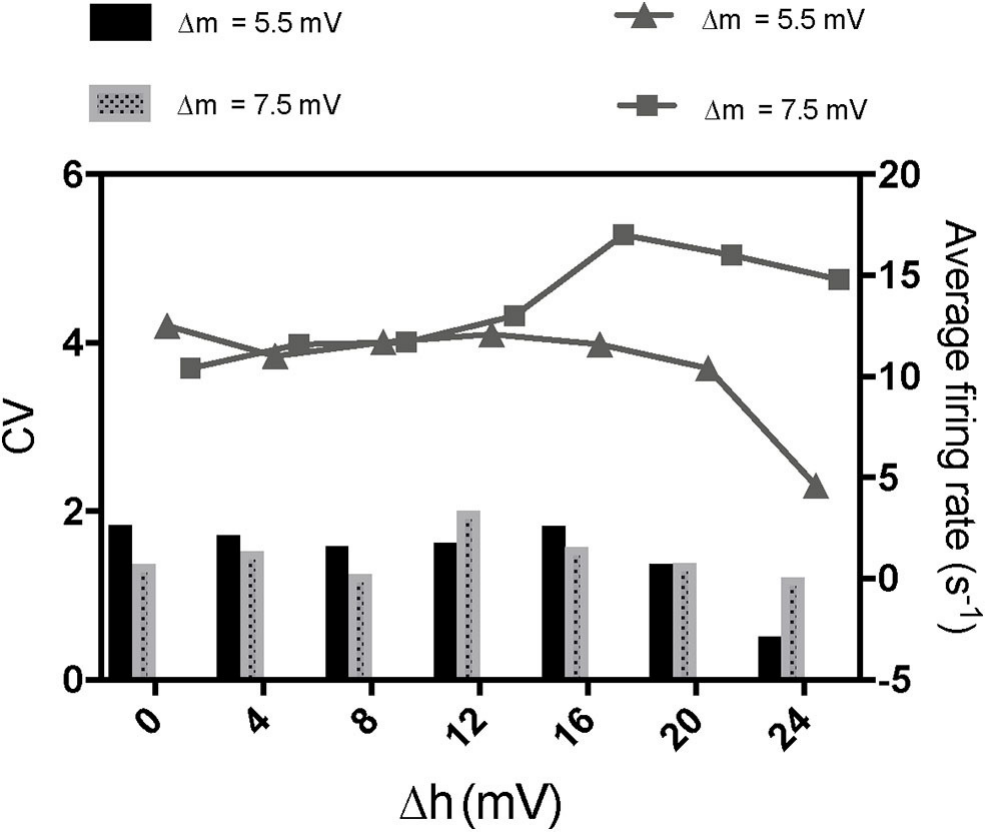
The effects of enhancement of Na_*V*_ inactivation on paradoxical excitement induced by acceleration of Na_*V*_ activation. The concentration of propofol was set as 5 times increase of GABA here.

To further investigate the mechanism of paradoxical action of the thalamocortical system, we manually removed the effects of propofol on Na_*V*_ in one cell population (Figure 8). PY cells still showed obvious irregular spiking after removing these effects in PY, RE, or IN cells. However, removing the effects in TC cells eliminated the abnormal activity of PY cells. We evaluated the cell spiking in the simulation (Figure 9), and found that in all cases with paradoxical activity, TC cells showed small and chaotic oscillations after the system was stable (Figure 9, bottom). Other cells were evoked with faster spiking with normal spiking shapes (Supplementary Figure 3). Within the Δh increased in system (Figure 10), for example at *IOG* value of 4 and Δh value of 4–20 mV, PY cells also showed paradoxical excitation and TC cells showed chaotic oscillation at Hopf bifurcation. However, with Δh value of 24 mV, the phenomena were eliminated, which was consistent with the two parameters bifurcation analysis that Hopf bifurcation was triggered with Δh less than 20.5 mV. Thus, these observations demonstrated that propofol accelerating Na_*V*_ activation and then evoking the Hopf bifurcation in TC cells was the main factor leading to a paradoxical excitation.

### 3.6. Enhancement of Inactivation Inhibits Activities of Thalamocortical Models

**Figure 11:**
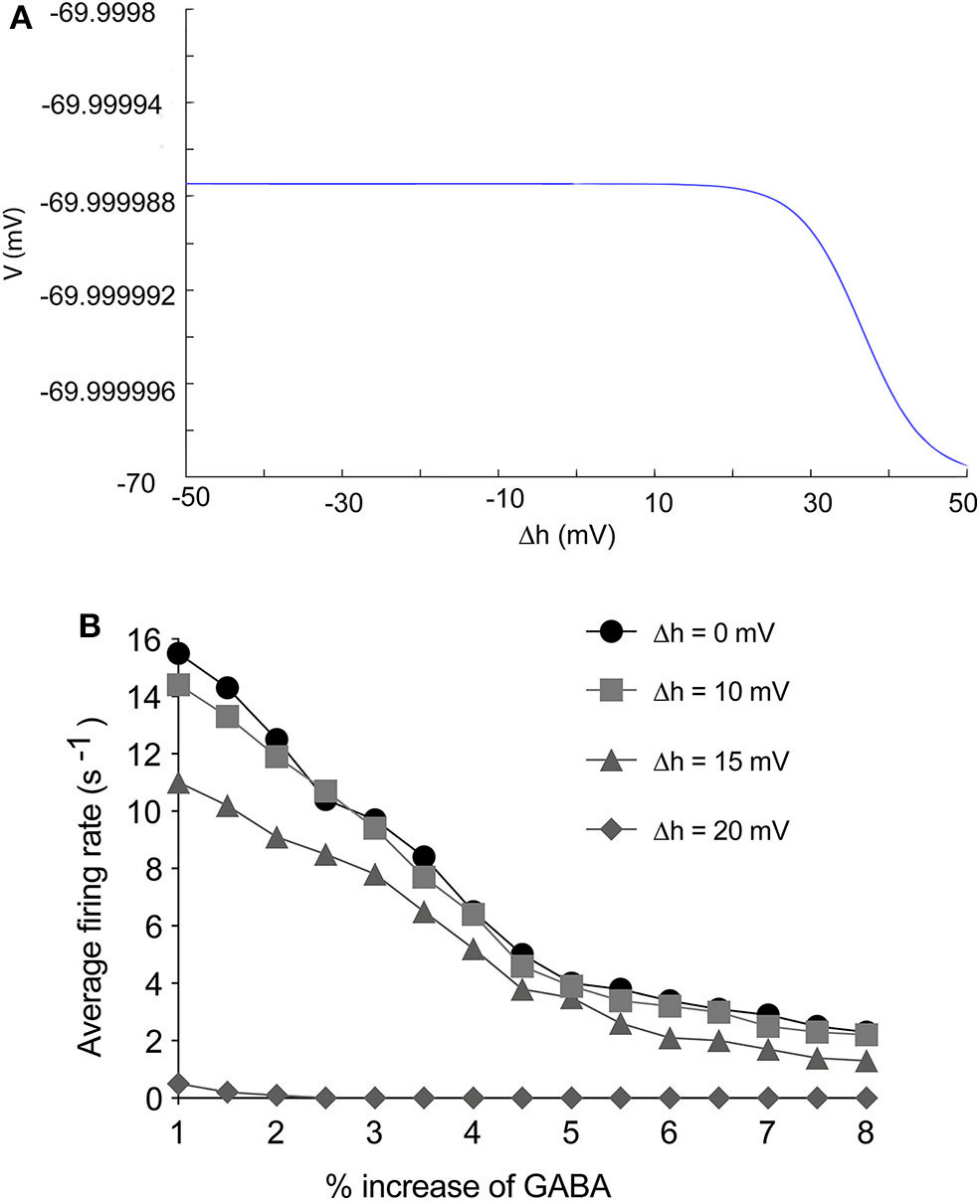
The effects of enhancement of Na_*V*_ inactivation on thalamocortical system. **(A)** The effects of Δh to cells stability. **(B)** The effects of Δh on firing rate of PY cells in thalamocortical system. The Δh was set as 0, 10, 15, 20 mV.

In our electrophysiology results, propofol mainly enhanced inactivation at high concentration, Thus, we tested the consequences that acceleration of inactivation might induce. Many researches have shown that acceleration of Na_*V*_ inactivation leads to inhibition of sodium channels (Wang and Strichartz, 2012; Tikhonov and Zhorov, 2017). Increasing Δh hyperpolarized cells implied that enhancement of Na_*V*_ inactivation tended to inhibit cells' oscillation (Figure 11). Therefore we studied the effect of Na_*V*_ inactivation on the thalamocortical model. The results showed that FR of PY cells did not decrease obviously with Δh value of 10 mV, compared with Δh value of 0. However, with Δh value of 15 mV, enhancement of Na_*V*_ inactivation significantly depressed the average firing rate of PY cells (Figure 11B). cellular activities were nearly depressed with the Δh value at 20 mV. This indicated that the effects of the drug on inactivation inhibited the system to an extent. Bupivacaine and lidocaine act as a pore blocker in sodium channels and show a strong shift in the inactivation of sodium channels with no effect on activation (Clarkson and Hondeghem, 1985). Many clinical trials have shown that application of lidocaine or bupivacaine during general anesthesia decreases the usage of general anesthetics for maintenance of anesthesia (Senturk et al., 2002; Bazin et al., 2018). Thus, our results indicated that local anesthetics decrease requirements of general anesthetics by enhancing Na_*V*_ inactivation.

## 4. Discussion

The mechanism of general anesthesia is still not completely understood. Mathematical models and computational simulations based on known biology are invaluable to uncover the dynamic mechanisms of the drugs involved. In our studies, we found that the followings: (i) Enhancement of activation is mainly induced by low concentrations of propofol, and acceleration of inactivation takes place in a dose-dependent manner. (ii) By altering gating parameters related to the equations of Na_*V*_ activation and inactivation based on the H-H model, we found that varying the shift of Na_*V*_ activation parameters enhanced cell activity by inducing Hopf bifurcation in neuron models with the essential channels. (iii) Low concentration of propofol accelerated Na_*V*_ activation in the thalamocortical system thereby inducing Hopf bifurcation in TC cells. This effect led to a suppression of normal burst in TC cells, resulting in the paradoxical excitation phenomenon; (iv) At high concentrations of propofol, a left shift in Na_*V*_ inactivation enhanced its inhibitory effects on brain activities.

### 4.1. Mammalian Na_*V*_ and NaChBac

Na_*V*_ initiates action potentials in most neurons. Thus far, nine different subtypes of sodium channels have been identified in mammalian cells (Goldin, 2001). However, according to protein alignment, these proteins share conserved sequences and show similar functions (de Lera Ruiz and Kraus, 2015; Catterall, 2017; Shen et al., 2017). Since Na_*V*_ 1.6 is the one most likely to initiate action potential (Hu et al., 2009), we adopted the Na_*V*_ 1.6 parameters in our network model. While mammalian Na_*V*_ is a single polypeptide consisting of four contiguous, homologous pore-forming domains, NaChBac is a tetrameric assembly of identical subunits from Bacillus halodurans (Ren et al., 2001). It has been discussed in many papers that mammalian Na_*V*_ and NaChBac have similar aminoacid sequences, and both of them have activation and inactivation gating mechanisms (Scheuer, 2014; Oelstrom and Chanda, 2016). Similar to NaChBac, activation and inactivation of mammalian Na_*V*_ are structurally independent that activation is related to S4 helices (Wisedchaisri et al., 2019) while inactivation is related to the short intracellular loop connecting homologous domains III and IV (Catterall et al., 2019). The inactivation of mammalian Na_*V*_ is more complicated, which contains fast inactivation and slow inactivation, with some studies even proposing a prolonged inactivation state (Navarro et al., 2020). Anesthetics mainly interact with the fast inactivation (Herold and Hemmings, 2012). As proposed by Lee (Lee et al., 2012) and Boiteux (Boiteux and Allen, 2016), the cytoplasmic DIII-IV linker and the cytoplasmic carboxyl terminus are likely the reason that mammalian Na_*V*_ inactivation begins with a fast inactivation. The cytoplasmic region occludes the pore by binding to the cytoplasmic linkers connecting segments 4 and 5 (S4-S5) in domains III and IV and the cytoplasmic end of the S6 segment in domain IV to close the channel (Goldin, 2003). The mechanisms of NaChBac inactivation have been proposed to occur in a manner homologous to pore rearrangement (Pavlov et al., 2005) or akin to activation gate “slippage” of the S6 segment (Shin et al., 2004). Despite these differences in the molecular dynamic mechanisms, studies have indicated that general anesthetics interact with S4-S5 linker and S6 segment to stabilize inactivation status in NaChBac (Barber et al., 2014; Kinde et al., 2016; Sand et al., 2017; Yang et al., 2018). Considering the similar sequences of mammalian Na_*V*_ and NaChBac in these segments (Scheuer, 2014; Oelstrom and Chanda, 2016), it is highly possible that anesthetics stabilizing the fast inactivation of mammalian Na_*V*_ and inactivation of NaChBac share similar molecular mechanisms. Since NaChBac conserves basic gating features but has slower kinetics, which makes it easier to discover dynamic mechanisms of sodium channels, it is widely used as a substitute for human Na_*V*_ to investigate Na_*V*_ structure and pharmacological properties (Barber et al., 2014; Yang et al., 2018). Moreover, *in vitro* studies have shown that mammalian Na_*V*_ (OuYang and Hemmings, 2007) and NaChBac (Sand et al., 2017) have similar pharmacological responses to similar concentration of isoflurane (0.82 and 0.8 mM). This indicates the applicability of NaChBac electrophysiology results to human Na_*V*_. In our electrophysiology experiments, propofol was observed to cause left shift activation, increasing with concentrations of 0.3–3 μM, and decreasing with concentrations from 3–30 μM, while inactivation increased with concentrations (Figure 2). According to clinical studies, the EC_50_ of propofol in human plasma is around 15 μM (Li et al., 2015), and maintenance is around 30 μM (Casati et al., 1999). Hence, We conclude that light anesthesia primarily accelerates Na_*V*_ activation, while deep anesthesia enhances Na_*V*_ inactivation.

### 4.2. Comparison of Our Model With Coupled Left-Shift of Na_*V*_ Channels

In our Na_*V*_ model, we simulated the effects of propofol on sodium channels by shifting the equations of activation and inactivation parameters, which were similar to previous publications (Boucher et al., 2012; Yu et al., 2012). These studies coupled activation and inactivation parameters by simultaneously inducing left shift, which showed that coupled left-shift of Na_*V*_ channels led to a Hopf bifurcation and induced spontaneous activities of injured node that may be a potential mechanism of the dysfunctional excitability of damaged axons (Boucher et al., 2012; Yu et al., 2012). However, the independence of activation and inactivation of sodium channel has been demonstrated in many publications. For example, a mutation at the T220 site in NaChBac—T220A—induces complete disappearance of inactivation process, while the channel still can be activated (Lee et al., 2012). In Wang's work, propofol binding to voltage-sensing domain to accelerate activation and S6 to enhance inactivation (Wang et al., 2018). Another study found that sevoflurane binds with S4–S5 linker sites to accelerate activation and selectivity filter to enhance inactivation (Barber et al., 2014). Furthermore, as shown in our electrophysiology results that propofol modulates activation and inactivation with different concentration-dependence, activation and inactivation are independent process. Thus, we separated these two gating parameters in our bifurcation analysis.

### 4.3. Bifurcation Analysis of H-H Model

Although Markov models (MM) have been widely used to investigate the kinetic changes and state-dependent features of the Na_*V*_ (Irvine et al., 1999; Chizhov et al., 2014; Yang et al., 2018), it has been reported that both the static and dynamic properties of the action potential were unchanged when the MM formulation was replaced with an homologous H-H model (Carbonell-Pascual et al., 2016). Further, H-H models have also been widely used in network simulations, wherein they were able to obtain electrophysiology recordings with many fewer calculations than those required by MM models (Pospischil et al., 2008). Thus, we adopted H-H models for further studies. Our bifurcation analysis showed that, left shift of Na_*V*_ activation and inactivation induced Hopf bifurcation when Δh is less than 20.5 mV, which was consistent with a previous study (Yu et al., 2012). However, by studying Na_*V*_ activation and inactivation separately, our study indicated that acceleration of activation was the main factor leading to oscillation, while enhancement of inactivation inhibited cells oscillation after a certain extent (Figures 5, 10). There has been considerable research into bifurcation theory in H-H dynamic systems (Doi et al., 2001; Zhang et al., 2014) to indicate that I_*app*_ leads to a bursting oscillations (Zhu and Wu, 2014). In the brain network, cells receive stimulus from other cells, whose effects on cellular dynamics are non-negligible. Our two parameters bifurcation analysis (Supplementary Figure 4) showed that I_*app*_ led to left shift of the Hopf bifurcation point. Since the external stimulus TC cell received is less than 0, it explains shifting of the Hopf bifurcation point in TC cells.

### 4.4. The Mechanism of Paradoxical Excitement Induced by General Anesthesia

Paradoxical excitation is a phenomenon observed in patients under light anesthesia. These patients typically exhibit increased talkativeness, loss of cooperation, disorientation, excessive movement, sexual hallucinations, agitation, and/or rage (Jeong et al., 2011). Because it is a fast process with varying severity and type of responses, currently there is no diagnostic criteria for paradoxical excitation responses, which makes it difficult to study the deep mechanism. Several clinical trails have attempted to study paradoxical excitation induced by general anesthesia (Jeong et al., 2011; Lee et al., 2019), and the results have shown that paradoxical excitation is usually observed in general anesthesia induced by propofol at low doses (4.5 and 8 μM) (Jeong et al., 2011). It was seen that paradoxical excitement is related to an increased oscillatory activity in the higher beta-frequency bands (12.5–25 Hz) and a decreased activity in the slower frequency bands (3.5–12.5 Hz) (McCarthy et al., 2008). Further, the M current in excitatory cells affects the synchronization of interneuron cells and could be the potential mechanism leading to the increasing beta-frequency bands. However, there were only cortical cells in their model, which did not explain why paradoxical excitement occurs selectively in several drugs, especially propofol. It is widely accepted that thalamus-cortex is involved with the consciousness (Redinbaugh et al., 2020). A thalamus-cortex model has been adopted to explore the mechanism of general anesthesia in many publications, and has shown that increased synchronization and decreased connectivity in thalamus-cortex are related to the loss of consciousness (Flores et al., 2017; Malekmohammadi et al., 2019). Thus, we constructed thalamocortical models to further understand the mechanism of general anesthesia.

Propofol was found to trigger seizure-like phenomenon at sedation concentration, which reminds us of the dynamic mechanism of seizures (Walder et al., 2002; Hickey et al., 2005). Based on our current understanding of paradoxical excitement, we still cannot exclude the possibility that seizure-like phenomenon is one type of paradoxical excitement. Several studies have shown that the mutations in activation or inactivation of sodium channel are one of the main mechanisms leading to dynamic changes in seizure (Lauxmann et al., 2013; Oliva et al., 2014). Therefore, we speculated whether the interaction between propofol and sodium channel would participate in paradoxical excitation. Our results showed that at low concentrations, increased dominant EEG frequencies were observed in the cortical cells in the models (Figure 7B), as was found in EEG manifestations of paradoxical excitation. Cellular activities also showed different modes, with less cells spiking but faster, which explained the abnormal behaviors apparent during paradoxical excitation (Figure 7C).

In paradoxical excitation, we observed that TC cell activity was suppressed, while other cells were activated. In the absence of the effects on sodium channels in TC cells, paradoxical excitation disappeared (Figure 8). This observation implies that the activity of TC is critical to paradoxical excitement. TC cell burst mode induced by activation of *I*_*h*_ and *I*_*T*_ has typically been associated with anesthetic states (Bezdudnaya et al., 2006; Zeldenrust et al., 2018), as shown in our simulation (Figure 9, top). However, our findings also showed that TC cells spiked abnormally in paradoxical excitation with small chaotic oscillations that were referred to irregular spiking of PY cells (Figure 8). Inactivation alone does not relate to the paradoxical excitement (Figure 11), and combining left shift of activation with inactivation still produced paradoxical excitement with a certain extent (Figure 10). Therefore, these results demonstrated that the effect of propofol on Na_*V*_ activation suppressed normal bursting of TC cells by triggering its Hopf bifurcation. As study indicated that anesthetics binding to NaChBac shows stated dependent, it is promising that developing Na_*V*_ inactivation-dependent anesthetics to avoid paradoxical excitement (Barber et al., 2014).

### 4.5. The Application of Sodium Channel Reduced the Requirement of General Anesthesia

The shift to inactivation would not significantly inhibit the paradoxical effect (Figure 10), similar to the findings obtained in Hans' clinical trials, which found that intravenous lidocaine infusion does not reduce the bispectral index-guided requirements of propofol during the induction process (Hans et al., 2010). Lidocaine is known to be the sodium channel blocker accelerating inactivation (Sheets et al., 2011). However, with additional shifts of inactivation, PY cells showed lower powered firing and frequency, indicating that propofol accelerating inactivation inhibited the central nervous system (Figure 11). Many clinical trials have shown that application of lidocaine or bupivacaine during general anesthesia decreases the usage of general anesthetics for maintenance of anesthesia (Senturk et al., 2002; Bazin et al., 2018). Multiple hypotheses have been proposed to explain why local anesthetics could reduce the requirement for general anesthetics, such as the inhibition of pain pathways. In our simulation, the reduction of presynaptic action potential amplitude by enhancement of Na_*V*_ inactivation could be one mechanism by which propofol inhibits CVS, and the application of local anesthetics leads to a lower requirement for general anesthetics (Liu et al., 2019).

### 4.6. Study Limitations

The limited number of cell types and synaptic connections in the model perhaps limited our ability to uncover the precise dynamic mechanisms of this system. Propofol also has effects on other receptors such as K_2_P or K_*v*_ channels (Stock et al., 2017, 2018), which are also essential for the membrane potential. However, as Barber described, the modulation of K_*v*_ channel is anesthetic-specific in that halothane, isoflurane, and desflurane inhibit it, while sevoflurane activates it (Barber, 2013). Studies have shown that propofol obviously inhibited cardiac potassium channel at 30 μM and pancreatic potassium channel at 50 μM (Yang et al., 2015; Kusunoki et al., 2019), which are greater than the concentrations used for light anesthesia. With complicated responses that general anesthetics to potassium channel, it still need more research in the future. We did not conduct *in vitro* experiments or assess brain slides to confirm our simulation results, because except for the equipment limitation in our laboratory, the pre-synaptic modification would be activated by propofol simultaneously, and the inward Cl^−^ would affect the recording results of the sodium channel activation (Liu et al., 2019). Additionally, the dynamic changes depicted in our studies are very fast and sensitive to the inputs from other cells. The minor external stimulus could affect dynamic changes. This is also the reason why animal models of paradoxical excitement do not yet exist. The mechanism requires further research and validation.

## 5. Conclusion

Based on electrophysiology results, we modified the Hodgkin–Huxley model to simulate the effects of propofol on Na_*V*_, and found that an acceleration in activation led to oscillations in individual cells induced by Hopf bifurcations. Using the widely accepted theory that propofol potentiates γ-GABAa receptor activity, we found that an acceleration in activation at low dosages of propofol led to chaotic oscillations of TC cells at Hopf bifurcation, instead of its normal bursts. The chaotic oscillations of TC cells resulted in paradoxical excitation in our thalamocortical model. An enhancement of inactivation at high dosages of propofol inhibited the system. These results are valuable for the understanding of potential mechanisms related to the complex manifestations of general anesthesia.

## Data Availability Statement

The original contributions presented in the study are included in the article/Supplementary Materials, further inquiries can be directed to the corresponding author/s.

## Author Contributions

JX performed the experiment, contributed significantly to analysis data, and wrote the manuscript. ZC helped construct the thalamocortical model and revise the manuscript. BY contributed to the conception of the study and analysis with constructive discussions. All authors contributed to the article and approved the submitted version.

## Conflict of Interest

The authors declare that the research was conducted in the absence of any commercial or financial relationships that could be construed as a potential conflict of interest.
