# Supplementary figures and images for: A Potential Mechanism of Sodium Channel Mediating the General Anesthesia Induced by Propofol
Source: Front Cell Neurosci. 2020 Dec 4;14:593050. doi: 10.3389/fncel.2020.593050 (PMC7746837; doi:10.3389/fncel.2020.593050)

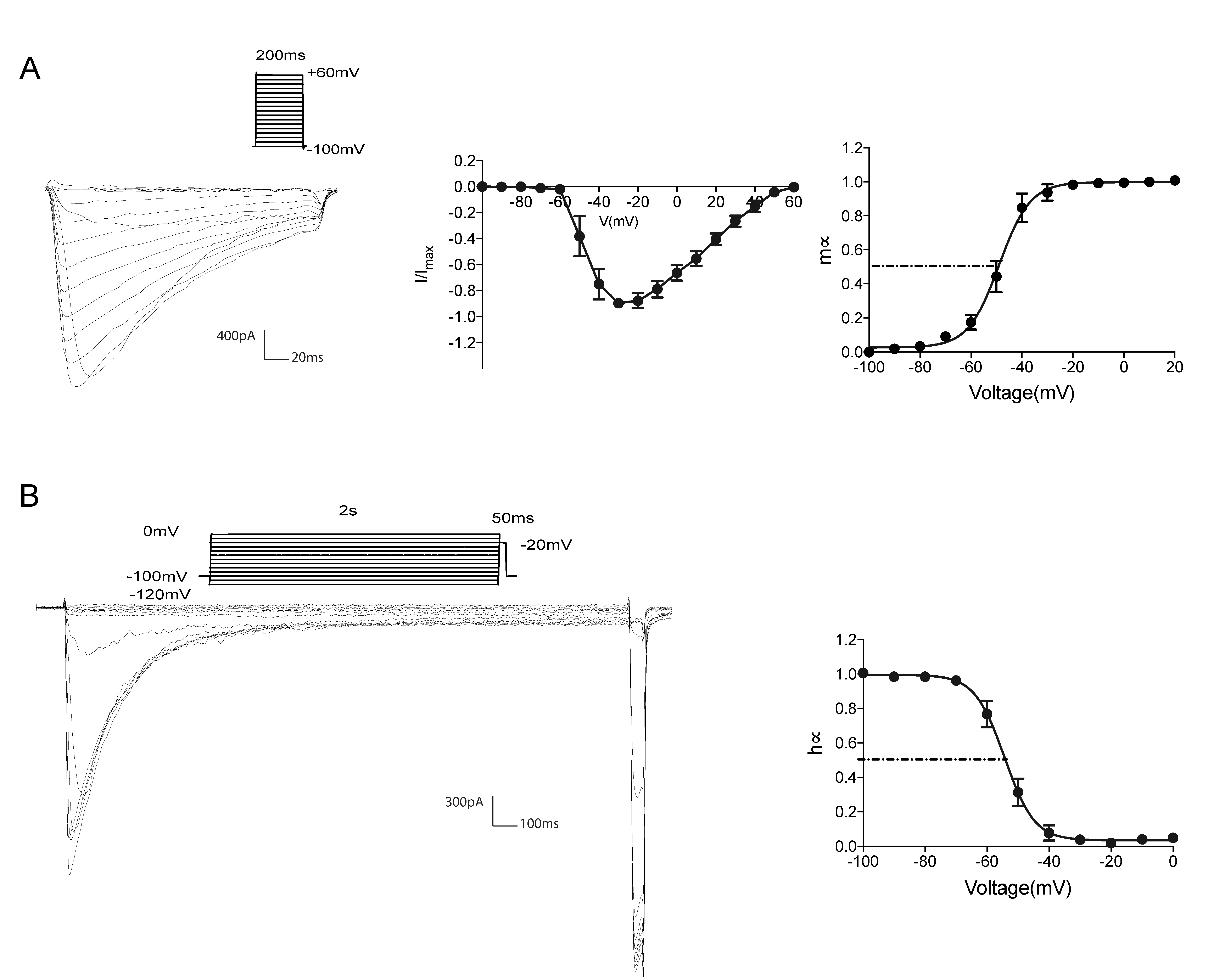

Supplement: Supplementary file 1 [file Image_1.tif]

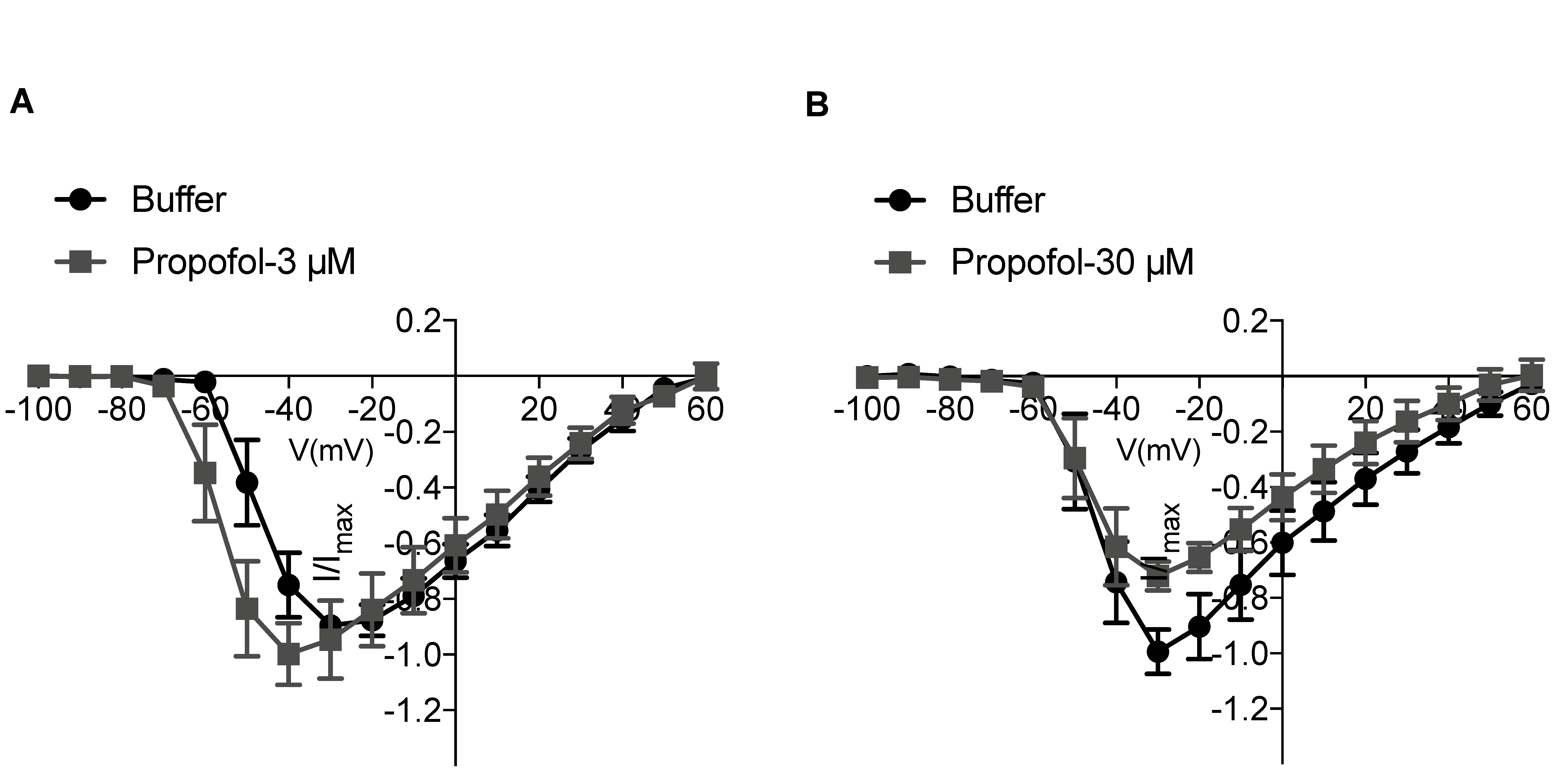

Supplement: Supplementary file 2 [file Image_2.tif]

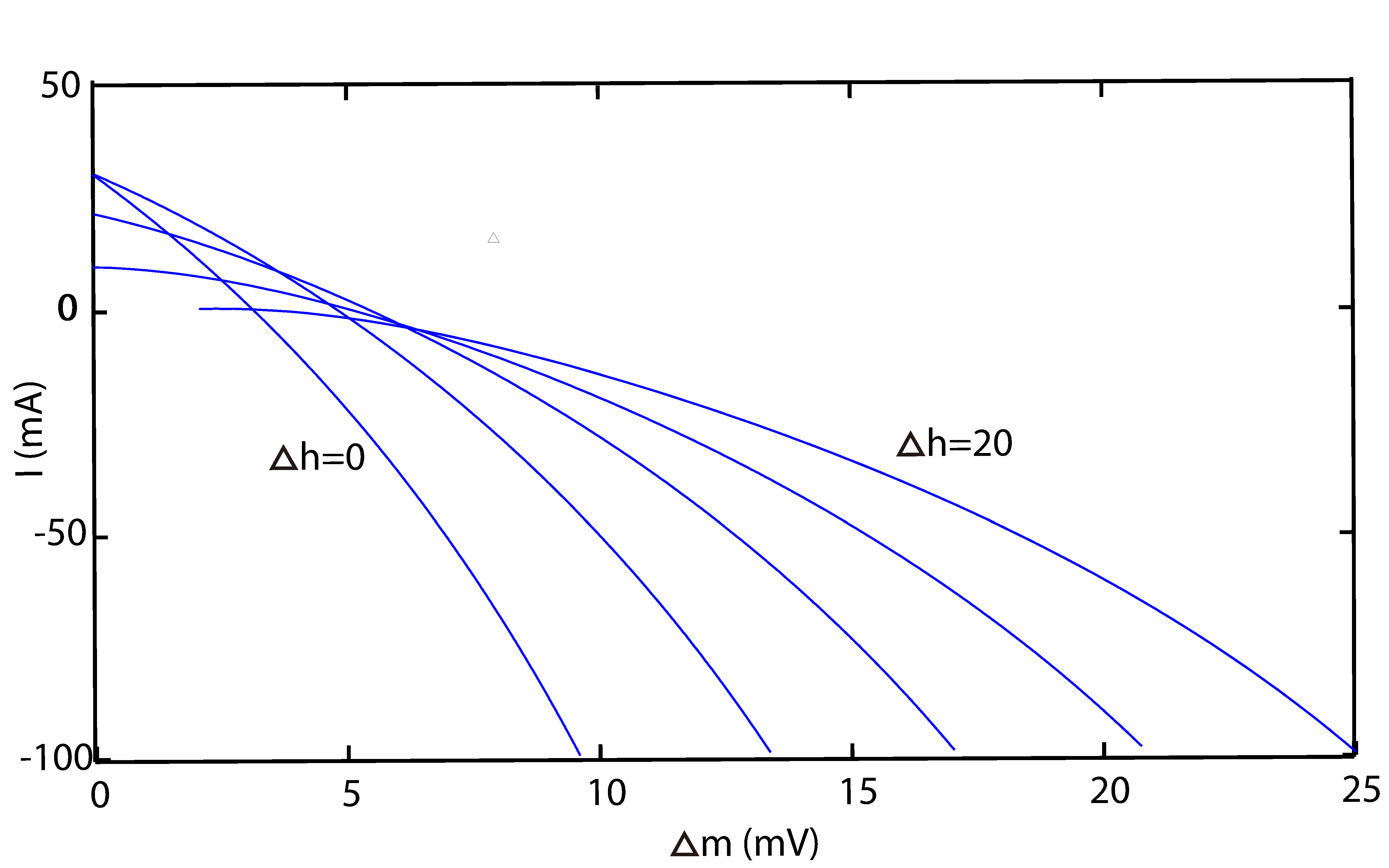

Supplement: Supplementary file 4 [file Image_4.tif]
